# Ketosis elevates plasma concentrations of D-β-hydroxybutyryl-phenylalanine (BHB-Phe) in humans

**DOI:** 10.1186/s12986-026-01172-7

**Published:** 2026-07-15

**Authors:** Mette Glavind Bülow Pedersen, Emaan Ghias, Maj Bangshaab, Thien Vinh Loung, Mads Bisgaard Bengtsen, Mads Vandsted Svart, Esben Søndergaard, Lars Christian Gormsen, Niels Møller, Mogens Johannsen, Nikolaj Rittig

**Affiliations:** 1 Steno Diabetes Center Aarhus, Aarhus University Hospital, Aarhus N, 8200 Denmark; 2 Medical/Steno Aarhus Research Laboratory, Department of Clinical Medicine, Aarhus University, Palle Juul-Jensens Blvd 11, Aarhus N, 8200 Denmark; 3 Department of Forensic Medicine, Aarhus University, Aarhus, Denmark; 4 Department of Internal Medicine, Horsens Regional Hospital, Horsens, Denmark; 5 Department of Endocrinology and Internal Medicine, Aarhus University Hospital, Aarhus, Denmark; 6 Department of Nuclear Medicine, Aarhus University Hospital, Aarhus, Denmark

**Keywords:** Beta-Hydroxybutyrate, D-β-hydroxybutyryl-phenylalanine, Ketogenic diet, Ketone supplementation, Amino acid conjugates, Appetite regulation

## Abstract

**Introduction:**

β-hydroxybutyrate (BHB), produced during ketosis, can conjugate with amino acids forming BHB-amino acids. D-BHB-phenylalanine (D-BHB-Phe) is the most abundant BHB-amino acid and has been shown to reduce appetite and induce weight loss in mice, but its function and regulation in humans remain unclear. The objective of this study was to determine whether D-BHB-Phe concentrations vary across different degrees and forms of ketosis in humans.

**Methods:**

Plasma D-BHB-Phe was measured in samples from four clinical cross-over trials (i) after 21 days on a ketogenic diet (*n* = 10), (ii) after oral consumption of 30 g ketone salt (KS) and 30 g ketone ester (KE) (*n* = 14), (iii) after oral KE at doses of 10, 20 and 40 g (*n* = 10) and (iv) after oral versus intravenous KS administration (*n* = 8).

**Results:**

After 21 days of ketogenic dieting, plasma D-BHB-Phe became detectable (median 2.1 nmol/L; range 0.9–19.9), compared with near-undetectable concentrations during the standard diet (median 0; range 0-0.9; *p* = 0.005). Oral KS and KE increased D-BHB-Phe compared with placebo (*intervention x time*, *p* < 0.001). D-BHB-Phe rose dose-dependently after 10 g, 20 g, and 40 g of KE (*intervention x time*, *p* < 0.001). Oral KS induced higher peak concentrations of D-BHB-Phe (15.6 ± 6.4 nmol/L) compared with iso-ketotic intravenous KS infusion, which elicited only a modest peak (1.5 ± 0.3 nmol/L; *intervention x time*, *p* < 0.001). A positive association between the D-BHB and D-BHB-Phe concentration was observed in pooled analyses but was not consistent across cohorts.

**Conclusion:**

D-BHB-Phe is inducible by ketosis in humans. Our findings suggest involvement of the splanchnic bed in its production and support further exploration of D-BHB-Phe’s role in appetite regulation and metabolic health.

**Clinical trial registrations:**

NCT05012748, NCT03935841, NCT05263401, NCT05581043.

**Supplementary Information:**

The online version contains supplementary material available at https://doi.org/10.1186/s12986-026-01172-7.

## Introduction

β-hydroxybutyrate (BHB), produced during low-insulin states such as fasting or ketogenic diets, serves as an energy substrate for vital organs and acts as a signaling molecule with diverse metabolic effects [1, 2]. Recently, an additional metabolic pathway for BHB was identified: the enzymatic BHB-ylation of free amino acids (N-β-hydroxybutyryl amino acids, BHB-amino acids) catalyzed by carnosine dipeptidase 2 (CNDP2) [3]. The CNDP2 also catalyzes lactylation of free amino acids [4], among which N-lactoyl-phenylalanine (Lac-Phe) is the most abundant. Lac-Phe has attracted particular interest due to its ability to suppress feeding and induce weight loss in mice models [5]. Similarly, D-BHB-Phe, the most abundant BHB-ylated amino acid, has been shown to have effects on appetite and energy balance in mice [3]. Both acute and chronic administration of D-BHB-Phe to diet-induced obese mice reduces food intake and body weight, likely through activation of brain regions involved in feeding behavior [3]. Ketosis induced by a ketogenic diet, fasting, or a ketone monoester supplement increases D-BHB and subsequently D-BHB-Phe levels in mice. Clinical studies have shown elevated D-BHB-Phe following ketone monoester supplementation [3, 6] but not following a 48-hour fast [7]. Beyond these findings, the physiological role of D-BHB-Phe remains incompletely understood, highlighting the need for further insight into the regulation of this ketone-derived metabolite. To address this gap, we analyzed plasma samples from four previously conducted clinical trials involving various ketosis-inducing interventions. Our study aimed to test the following hypotheses:

1. A 3-week ketogenic diet will increase D-BHB-Phe concentration in humans.
2. Oral administration of ketone supplements, both ketone ester (KE) and ketone salt (KS), will increase D-BHB-Phe concentrations.
3. Increasing the circulating concentrations of D-BHB will result in increasing concentrations of D-BHB-Phe resulting in a positive correlation between circulating concentrations of D-BHB and D-BHB-Phe.
4. Oral BHB administration elicits a more pronounced D-BHB-Phe response than isoketonic intravenous BHB administration, similarly to previous findings for L-Lac-Phe [8].

To evaluate these hypotheses, we used plasma samples from four previously conducted clinical cross-over trials to quantify D-BHB-Phe concentrations and compare them with corresponding D-BHB levels.

## Methods

### Design and volunteers

The data presented in this study originate from four separate studies conducted at the Medical/Steno Aarhus Research Laboratory, Aarhus University, and the Department of Nuclear Medicine and PET Center, Aarhus University Hospital, Denmark, between 2019 and 2023. Key aspects of the trial designs and participant characteristics are summarized below for convenience. All four studies were approved by the Central Denmark Regional Scientific Ethics Committee and registered on www.clinicaltrials.gov. All participants gave oral and written informed consent to study participation.

- Cohort I [9] (NCT05012748): A randomized, controlled, crossover trial comparing a three-week ketogenic diet with a three-week standard diet, separated by a one-week washout. Each intervention period was followed by a study day, during which the utilized plasma samples were obtained. The study initially included 12 participants; however, one participant was excluded from the study due to a severe migraine on the first study day, and another lacked additional available plasma samples for the current study. Consequently, plasma samples from ten individuals were included in the current analysis (Supplementary Fig. 1). Participants were aged 50–70 years with a BMI of 28–40 kg/m². Experiments were conducted between September 2021 and August 2022, with no additional follow-up. On study days, the BHB concentration was measured as the total blood DL-BHB concentration using hydro-philic interaction liquid chromatography-tandem mass spectrometry. (HILIC-MS/MS). Because human L-BHB production is minimal, the measured values are assumed to reflect D-BHB. For consistency, results from this trial are presented as D-BHB. The plasma D-BHB-Phe concentration was measured at baseline (0 min) on each study day.
- Cohort II [10] (NCT05263401): A randomized-single blinded cross-over study investigating the effects of sodium-DL-β-hydroxybutyrate (KS), ketone ester (D-β-hydroxybutyrate-D-1,3-butanediol, KE), and placebo given as a premeal 30 min before a mixed meal test (MMT) in individuals with type-2 diabetes. Participants were studied on three separate occasions. One after ingestion of 30 g of KS (Gold Biotechnology, MO, USA), one after ingestion of 30 g of KE (KetoneAid, VA, USA), and one after ingestion of placebo (tap water with Bitrex ^®^ (Veranova, Edinburgh, UK), in random order. There was a minimum washout period of one week between interventions. A total of 14 individuals with type-2 diabetes were included (Supplementary Fig. 2). All participants were treated with either lifestyle/dietary interventions or metformin monotherapy only. Metformin treatment was discontinued five days before the study days. Study days took place between January 2022 and September 2022. There was no additional follow-up. Blood D-BHB concentrations have been reported previously [10] and were measured using point-of-care ketone test strips (Abbott Diabetes Care ltd., Oxon, UK). The plasma D-BHB-Phe concentration was measured at − 30, 0, 30, and 90 min relative to MMT administration, corresponding to 0 (baseline), 30, 60, and 120 min after ingestion of the KE/KS/placebo intervention.
- Cohort III [10] (NCT05581043): A randomized, single-blind, crossover trial including ten individuals with type 2 diabetes managed with either metformin monotherapy or dietary/lifestyle interventions (Supplementary Fig. 3). Participants attended six separate study visits, each separated by a minimum washout period of five days. Participants were randomly assigned to the following pre-meal interventions: (1) 0 g KE (KetoneAid, VA, USA),, (2) 10 g KE, (3) 20 g KE, or (4) 40 g KE administered 30 min before a 75 g oral glucose tolerance test (OGTT); (5) 20 g KE administered immediately before the 75 g OGTT; or (6) 20 g KE administered 60 min before the 75 g OGTT. For the present analysis, only study days in which participants received 0, 10, 20, or 40 g KE 30 min before the OGTT were included. Study days took place between February 2023 and December 2023. There was no additional follow-up. Blood D-BHB concentrations have been reported previously [10] and were measured using point-of-care ketone test strips (Abbott Diabetes Care ltd., Oxon, UK). Plasma D-BHB-Phe concentration was measured at − 30-, 0-, 30-, and 90-minutes relative to OGTT administration, corresponding to 0 (baseline), 30, 60, and 120 min after ingestion of the KE intervention.
- Cohort IV [11] (NCT03935841): A non-randomized controlled trial investigating the effects of oral versus iso-ketotic intravenous ketone salt infusion. The study consisted of two separate study days. On day one, participants received 30 g oral ketone as a ketone salt (sodium-D/L-3-hydroxybutyrate, KS (Gold Biotechnology, MO, USA)); on day two, they received an isoketonemic intravenous KS infusion. There was a minimum washout period of 2 days between interventions. A total of eight healthy male participants were included (Supplementary Fig. 4). Experiments were conducted between June and October 2019. Blood D-BHB concentrations have been reported previously [11] and were measured using point-of-care ketone test strips (Abbott Diabetes Care ltd., Oxon, UK). Plasma D-BHB-Phe concentration was measured at 0, 30, 60, and 120 min on each study day.

### Blood samples

In all four cohorts, blood samples were placed on ice, centrifuged, and frozen at -20 °C immediately after sampling and stored at -80 °C for batch analyses. Plasma used in the current study had been stored at -80 °C since completion of the original studies.

### Liquid chromatography tandem mass spectroscopy

Targeted quantification of D-BHB-Phe in plasma was performed by liquid chromatography-tandem mass spectrometry (LC-MS/MS) using multiple reaction monitoring in negative ionization mode. Protein precipitation and metabolite extraction were carried out using methanol containing an isotopically labeled internal standard, followed by centrifugation, evaporation, and reconstitution in aqueous formic acid. Analytes were separated on a reversed-phase C18 column using a 15-min gradient and detected on a QTRAP 6500 + mass spectrometer. Data acquisition and processing were performed using Analyst and MultiQuant software, and analyte peaks were normalized to the internal standard. Quantification was achieved using a seven-point calibration curve. Comprehensive LC-MS/MS settings, transitions, and sample preparation details are provided in the Supplementary Methods.

### Statistics

Data from Cohort I were analyzed using the Wilcoxon rank sum test, as differences between interventions did not follow a normal distribution. In Cohorts II-IV, the effects of interventions on the D-BHB-Phe concentrations over time (from baseline to 120 min) were evaluated using a linear mixed-effects model with repeated measures. The model included intervention, time, and their interaction as fixed effects and a random intercept for each participant. Given the small sample size, variance components were estimated using restricted maximum likelihood (REML), and the Kenward-Roger method was applied to adjust standard errors and degrees of freedom. In Cohort II, the model residuals showed a distinct flooring effect that could not be corrected using a logarithmic transformation of the outcome variable. As a sensitivity analysis, the same mixed-effects model was refitted using cluster-robust variance estimation at the participant level. Incremental area under the curve (iAUC) data were analyzed using similar mixed-effects models, with intervention as a fixed effect and a random intercept for each participant. Pairwise comparisons were performed to estimate differences between intervention groups, with Bonferroni correction applied to adjust for multiple comparisons. As an additional sensitivity analysis in Cohort II, individual iAUC values were compared between interventions using a Wilcoxon signed-rank test.

Exploratory correlation analyses were conducted to examine the association between D-BHB-Phe and D-BHB concentrations. In Cohort I, Spearman’s rank correlation was applied because neither D-BHB-Phe nor D-BHB concentrations were normally distributed, and an extreme outlier was present. In Cohorts II-IV, 60-minute D-BHB and D-BHB-Phe concentrations, corresponding to peak D-BHB-Phe, were correlated. Pearson’s correlation was used when both variables within an intervention were normally distributed; otherwise, Spearman’s rank correlation was applied. Normality was assessed using the Shapiro-Wilk test. To account for multiple testing, p-values from these correlation analyses were adjusted for multiple comparisons within each cohort using the Holm-Bonferroni method.

As an exploratory analysis prompted by visual inspection of the correlation plots and with an aim to further elucidate the relationship between D-BHB-Phe and D-BHB concentrations, the metabolite concentrations measured at 60 min in Cohort III were examined using a linear mixed-effects model with repeated measures. The model specified D-BHB-Phe as the outcome, included D-BHB concentration and intervention as fixed effects, and a random intercept for each participant.

Lastly, exploratory correlation and regression analyses were performed on aggregated data from interventions involving orally induced ketosis (Cohorts II-IV). To ensure independence of observations, mean D-BHB and D-BHB-Phe concentrations across all timepoints (0–120 min) and, for Cohorts II and III, across all interventions with ketone supplements were calculated for each participant. Linearity and homoscedasticity were assessed visually using a residual-versus-fitted plot, and normality of residuals was examined visually using a Q-Q plot.

A two-sided P value < 0.05 was considered statistically significant. Results in the text are expressed as mean ± SD or median (range). Graph data are modelled as mean ± SEM, mean ± SD, or median (75% percentiles) as indicated in the figure legends. Power calculations were omitted as these data were derived from four pre-existing clinical trials. All analyses were performed using Stata 14 (Stata Corp LP, College Station, Texas, USA), and graphs were modeled in GraphPad Prism 10 (GraphPad Software, San Diego, California, USA).

## Results

Cohort baseline characteristics are summarized in Table 1.

**Table 1:** Participant characteristics

	Cohort I	Cohort II	Cohort III	Cohort IV
Number of participants	10	14	10	8
Age (years)	54.5 (50–68)	54.5 (39–74)	57 (54–71)	24 (range, 21–34)
BMI (kg/m^2)^	31.3 (28–41)	30.5 (27–34)	26.5 (23–33)	25 (range, 20–30)
Female/Male (%)	50/50	50/50	30/70	0/100
Diabetes duration, years	N/A	2.3 (1–17)	4 (1–15)	N/A
Metformin treatment (%)	0	87	60	0
Statin treatment (%)	0	43	20	0
HbA1c (mmol/mol)	37 (25–47)	50 (41–74)	43.5 (31–54)	32.5 (27–35)
LDL cholesterol (mmol/L)	3.6 (2-4.2)	2.2 (1.7–2.9)	N/Av.	N/Av.
HDL cholesterol (mmol/L)	1.5 (1.1–1.9)	1.2 (0.7–1.9)	N/Av.	N/Av.
Total cholesterol (mmol/L)	5.8 (3.7–6.4)	4.5 (3.4–6.4)	N/Av.	N/Av.

BMI: Body mass index. N/A: not applicable. N/Av.; not available. Age, BMI, diabetes duration, HbA1c, LDL-, HDL and total cholesterol are shown as median (range).

### Endogenous ketosis: the ketogenic diet

**Fig. 1:**
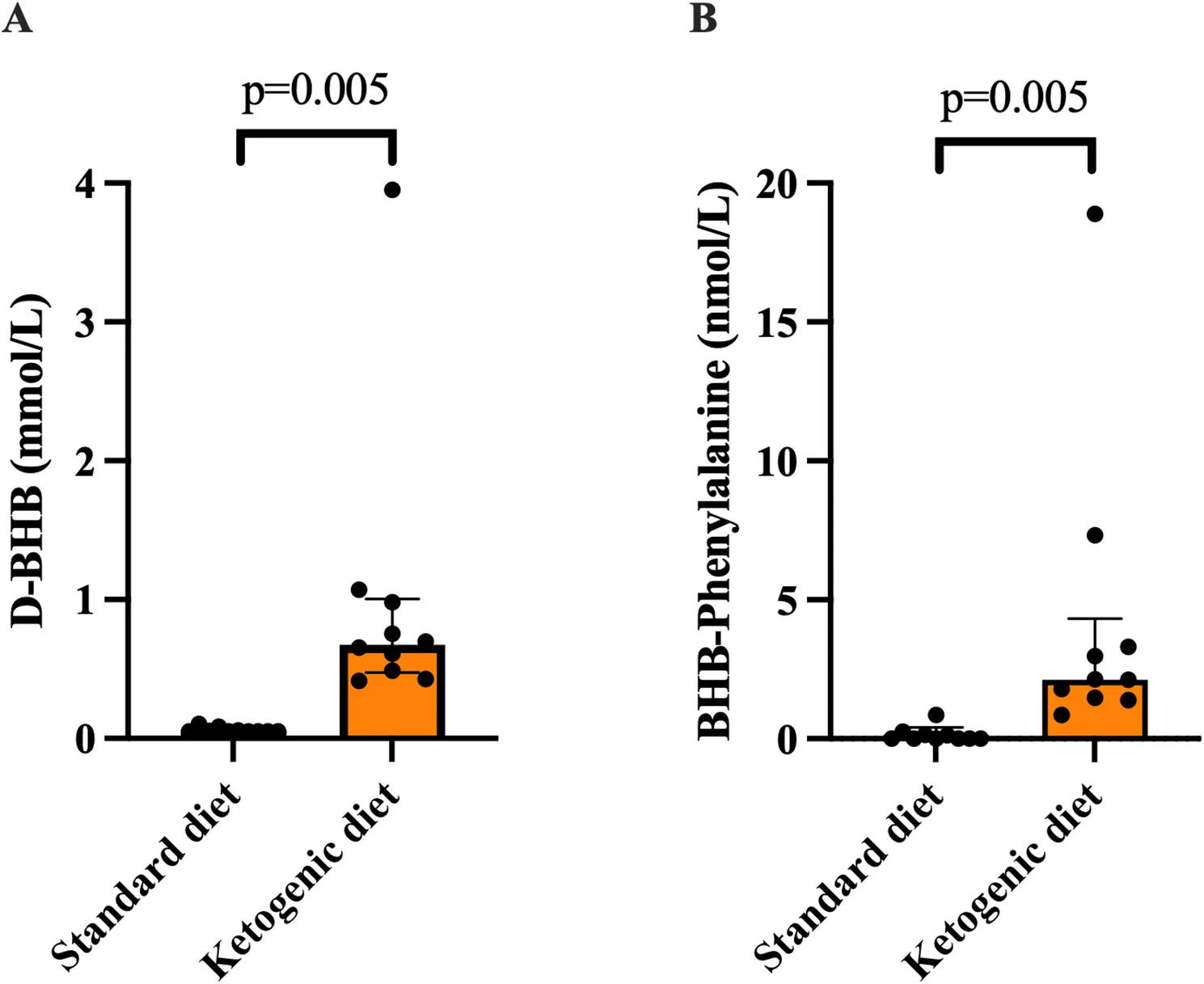
Effects of the ketogenic diet on D-BHB-Phe and D-BHB concentrations (*n* = 10). **(A)** Blood D-BHB concentrations after 21 days on a standard diet and 21 days on a ketogenic diet. Data are presented as median with 75th percentile. Wilcoxon signed-rank test: *p* = 0.005. **(B)** Plasma D-BHB-phenylalanine concentrations after 21 days on a standard diet and 21 days on a ketogenic diet. Data are presented as median with 75th percentile. Wilcoxon signed-rank test: *p* = 0.005

As previously reported, D-BHB concentrations were a median of 0.7 mmol/L (range: 0.4-4.0 mmol/L) after 21 days on the ketogenic diet and 0.05 mmol/L (range: 0.05–0.1 mmol/L) after 21 days on a standard diet (Fig. 1A) [9]. The median D-BHB-Phe concentration was 2.1 nmol/L (range: 0.9–18.9 nmol/L) after 21 days on the ketogenic diet and 0 nmol/L (range: 0-0.9 nmol/L) following 21 days on the standard diet (Fig. 1B). The D-BHB-Phe concentration was significantly higher on the ketogenic diet (Wilcoxon signed-rank test, *p* = 0.005, Fig. 1B).

### Exogenous ketosis: oral ketone ester versus oral ketone salt

**Fig. 2:**
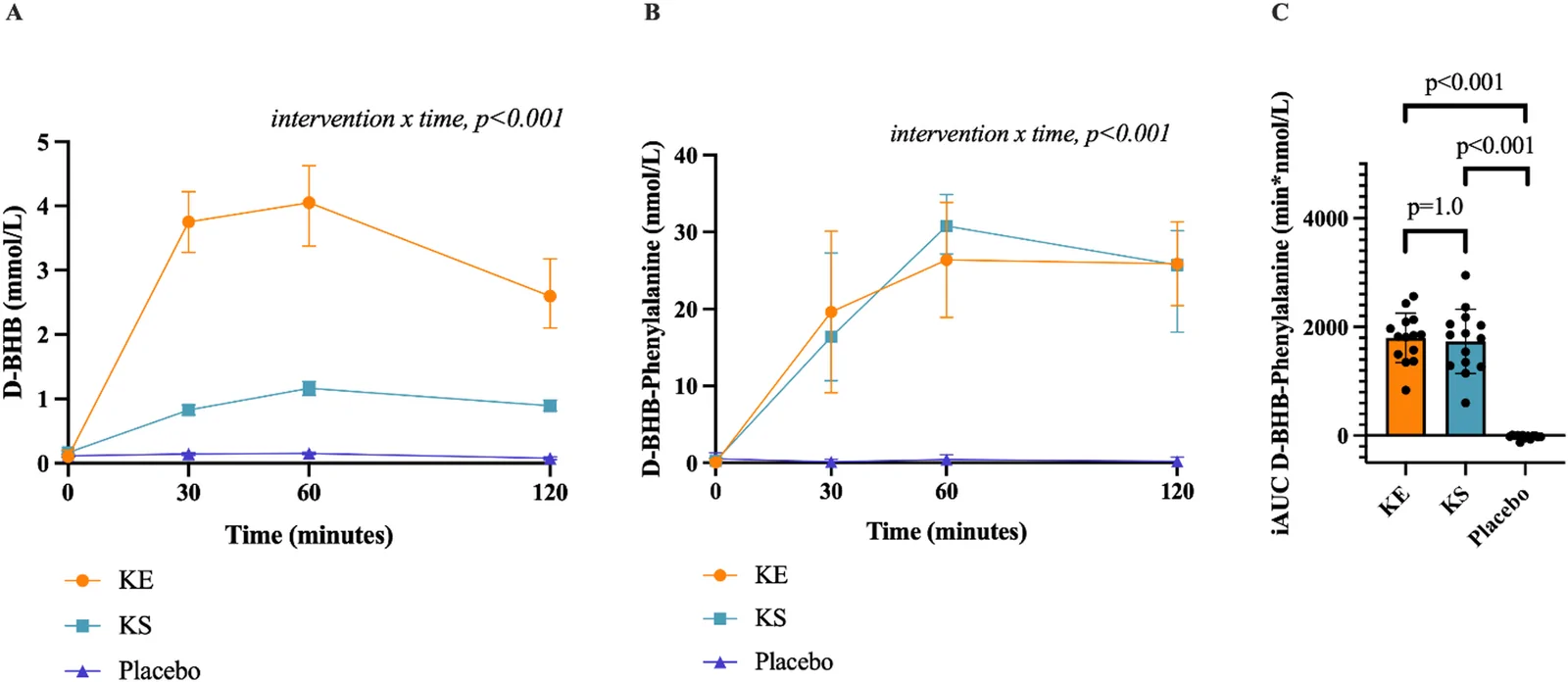
Effects of oral ketone ester and ketone salt on D-BHB and D-BHB-Phe (*n* = 14). **(A)** Mean Blood concentrations of D-BHB following oral KE (orange), KS (teal) or placebo (blue) administration, as published previously [10] **(B)** Plasma concentrations of D-BHB-Phenylalanine following oral KE (orange), KS (teal) or placebo (blue) administration **(C)** Incremental area under the curve (iAUC) of D-BHB-Phenylalanine 120 min after KE (orange), KS (teal) and placebo (blue) administration. Data in (**A**) and (**B**) are presented as mean ± SEM. Data in (C) are presented as mean ± SD. P-values shown in (**A**) and (**B**) corresponds to the *intervention x time* interaction from the mixed effects model. P-values in (**C**) reflect pairwise comparisons based on a mixed-effects model with Bonferroni correction for multiple comparisons. D-BHB: D-β-hydroxybutyrate. KE: Ketone ester. KS: Ketone salt. iAUC: Incremental area under the curve

As previously reported, D-BHB concentration peaked at significantly higher concentrations after KE ingestion compared with KS ingestion (Fig. 2A) [10]. Both KE and KS ingestion increased the D-BHB-Phe concentration compared with placebo (Fig. 2B, *intervention x time*, *p* < 0.001). The effect remained significant (*intervention x time*, *p* < 0.001) in the mixed-effects model with cluster-robust variance estimation. D-BHB-Phe peaked at a mean concentration of 26.4 ± 7.5 nmol/L after KE and 29.6 ± 9.2 nmol/L after KS, with no significant difference in iAUC for D-BHB-Phe between KE and KS (pairwise comparison, *p* = 1.0, Fig. 2C). Wilcoxon signed-rank test of iAUC values yielded the same qualitative conclusion with no difference between KE and KS and significantly higher responses compared with placebo (data not shown).

### Exogenous ketosis: dose-response relationship

**Fig. 3:**
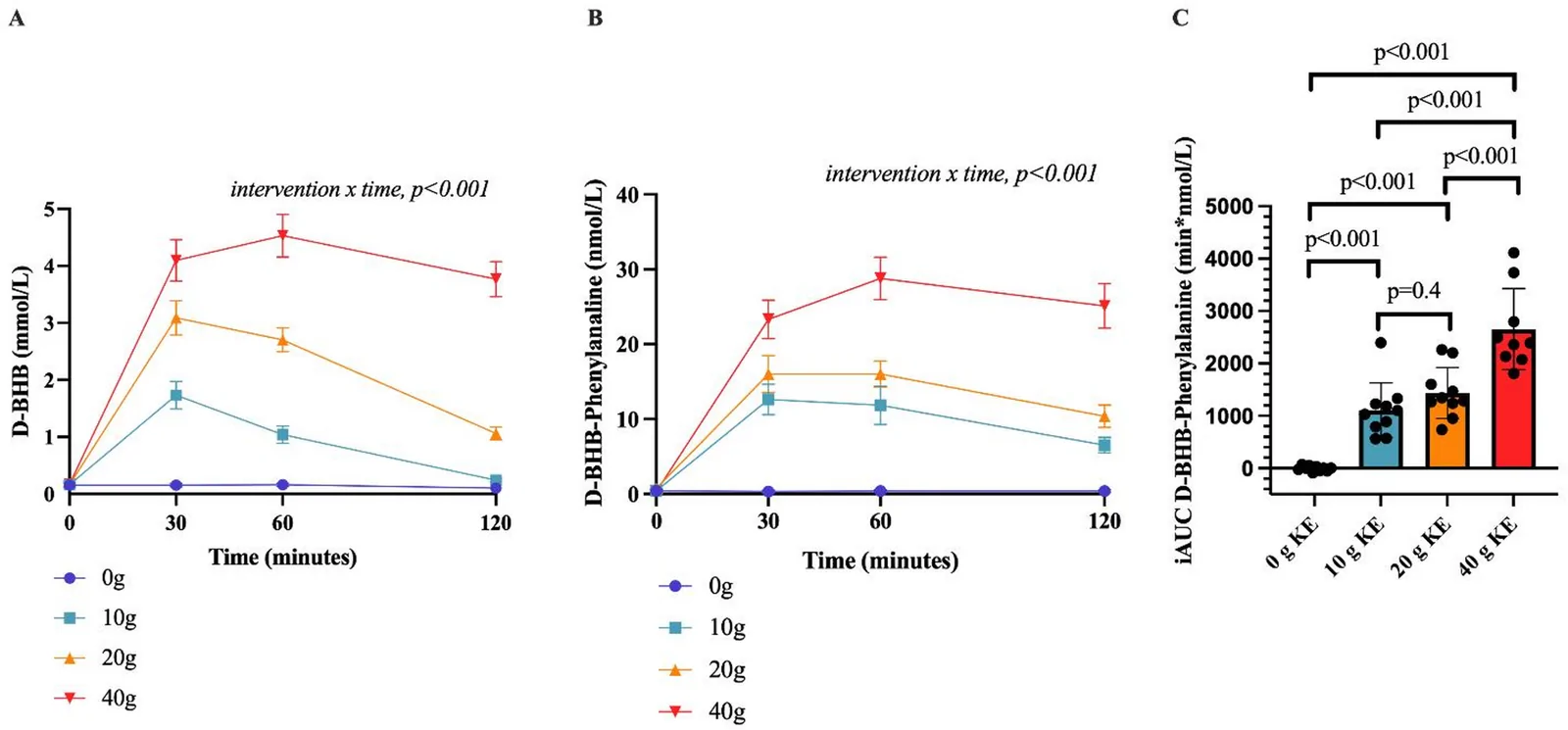
Effects of increasing dosages of ketone ester on D-BHB and D-BHB-Phe (*n* = 10). (**A**) Blood concentrations of D-BHB following 0 g of KE (blue), 10 g of KE (teal), 20 g of KE (orange) and 40 g of KE (red), as published previously [10] (**B**) Plasma concentrations of D-BHB-Phenylalanine following 0 g of KE (blue), 10 g of KE (teal), 20 g of KE (orange) and 40 g of KE (red) (**C**) Incremental area under the curve (iAUC) of D-BHB-Phenylalanine 120 min after 0 g of KE (blue), 10 g of KE (teal), 20 g of KE (orange) and 40 g of KE (red). Data in (**A**) and (**B**) are presented as mean ± SEM. Data in (**C**) are presented as mean ± SD. P-values shown in (**A**) and (**B**) corresponds to the *intervention x time* interaction from the mixed effects model. P-values in (**C**) reflect pairwise comparisons based on a mixed-effects model with Bonferroni correction for multiple comparisons. D-BHB: D-β-hydroxybutyrate. KE: Ketone ester. KS: Ketone salt. iAUC: Incremental area under the curve

As previously reported, oral administration of 10 g, 20 g, and 40 g KE increased D-BHB to mean peak values of 1.8 ± 0.7 mmol/L, 3.3 ± 0.8 mmol/L, and 4.9 ± 1.1 mmol/L, respectively (Fig. 3A) [10]. The D-BHB-Phe concentration increased after KE administration, with the most pronounced increase after 40 g KE (Fig. 3B, *intervention x time*, *p* < 0.001). Peak D-BHB-Phe concentrations were 12.5 ± 8.0 nmol/L following 10 g KE, 16.1 ± 5.6 nmol/L following 20 g KE, and 28.7 ± 9.0 nmol/L following 40 g KE. The iAUC for D-BHB-Phe increased in a similar dose-dependent manner, although values did not differ between 10 g and 20 g of KE (Fig. 3C).

### Exogenous ketosis: oral versus intravenous administration

**Fig. 4:**
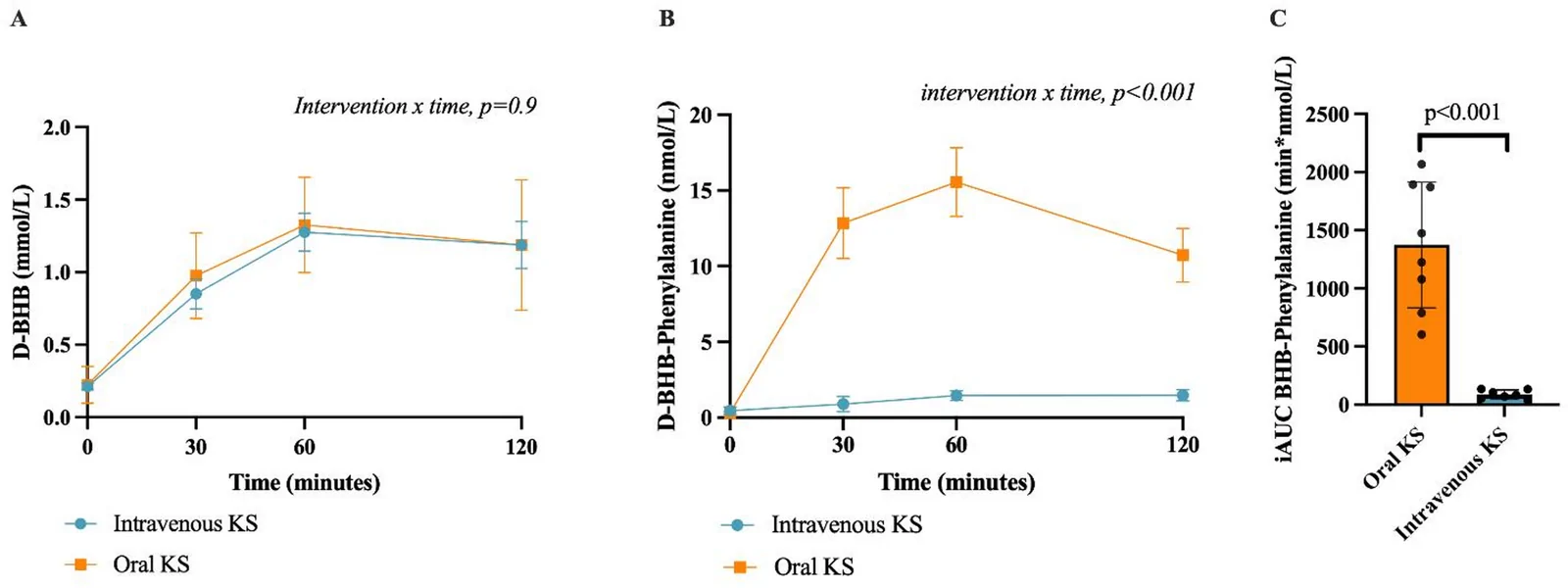
Effects of oral- and intravenous administration of ketone salt on D-BHB and D-BHB-Phe concentrations (*n* = 8). (**A**) Blood concentrations of D-BHB following oral (orange) and intravenous (teal) administration of KS, as published previously [11] (**B**) Plasma concentrations of D-BHB-Phenylalanine following oral (orange) and intravenous (teal) administration of KS (**C**) incremental area under the curve (iAUC) of D-BHB-Phenylalanine 120 min after oral (orange) and intravenous (teal) KS administration Data in (**A**) and (**B**) are presented as mean ± SEM. Data in (**C**) are presented as mean ± SD. P-values shown in (**A**) and (**B**) corresponds to the *intervention x time* interaction from the mixed effects model. P-values in (**C**) reflect pairwise comparisons based on a mixed-effects model with Bonferroni correction for multiple comparisons. D-BHB: D*-*β-hydroxybutyrate. KE: Ketone ester. KS: Ketone salt. iAUC: Incremental area under the curve

As previously reported and as a premise of the study design, the D-BHB concentration was similar between the two study days (Fig. 4A, *intervention x time*, *p* = 0.9) [11]. Oral KS administration resulted in a greater increase in D-BHB-Phe compared with intravenous KS (Fig. 4B, *intervention x time*, *p* < 0.001) peaking at 15.6 ± 6.4 nmol/L versus 1.5 ± 0.3 nmol/L, respectively.

### Relationship between D-BHB and D-BHB-Phe concentrations

**Fig. 5:**
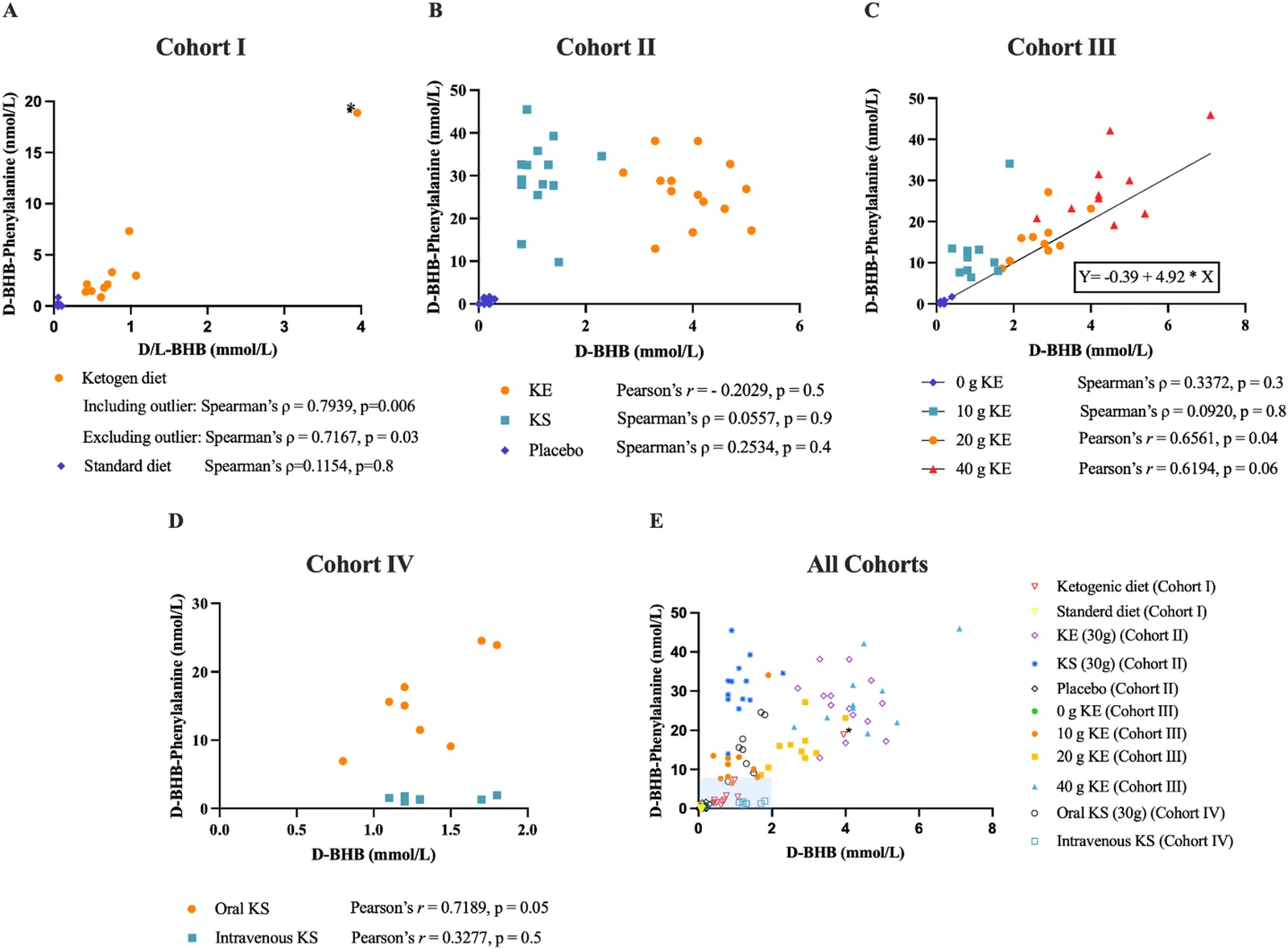
Correlations between the D-BHB and D-BHB-Phe concentrations. **(A)** Correlation between D-BHB and D-BHB-phenylalanine after 21 days on a ketogenic diet. Asterisk indicates an extreme outlier. **(B)** Correlation between D-BHB and D-BHB-phenylalanine concentrations measured at 60 min after oral KE (orange) and KS (teal) administration. **(C)** Correlation between D-BHB and D-BHB-phenylalanine concentrations measured at 60 min after 10 g of KE (teal), 20 g of KE (orange) and 40 g of KE (red). The regression line is based on a linear mixed-effects model and shows the fixed-effects association between D-BHB and D-BHB-phenylalanine (regression equation shown in the figure: *Y = − 0.39 + 4.92× X*). **(D)** Correlation between D-BHB and D-BHB-phenylalanine concentrations measured at 60 min after oral (orange) and intravenous (teal) administration of KS. **(E)** Plotted values of D-BHB and D-BHB-phenylalanine measures at baseline (Cohort I) or at time 60 min (Cohorts II-IV) across all four cohorts. Observations from placebo/control interventions, intravenous ketone infusion, and endogenous ketosis (all conditions in which ketones are not present in the gut) are shown in the blue box; the asterisk marks one outlier from Cohort I. Displayed p-values are unadjusted. Statistical significance after Holm-Bonferroni correction is reported in the Results text. D-BHB: D-β**-**hydroxybutyrate KE: Ketone ester. KS: Ketone salt

In Cohort I, D-BHB and D-BHB-Phe concentrations after 21 days on a ketogenic diet showed a strong positive correlation (Fig. 5A; Spearman’s ρ = 0.7934, unadjusted *p* = 0.006); this correlation remained statistically significant after Holm-Bonferroni correction. However, in a sensitivity analysis excluding an extreme outlier, the correlation was attenuated (Spearman’s ρ = 0.7167, unadjusted *p* = 0.03) and did not remain statistically significant after correction. There was no correlation after the standard diet. In Cohort II, no significant correlation was observed between D-BHB and D-BHB-Phe concentrations (KE: Pearson’s *r* = − 0.20, *p* = 0.5; KS: Spearman’s ρ = 0.06, unadjusted *p* = 0.9; Placebo: Spearman’s ρ = 0.25, unadjusted *p* = 0.4; Fig. 5B). Results were unchanged after Holm-Bonferroni correction. In Cohort III, the mixed model analysis showed a positive association between D-BHB-Phe and D-BHB across KE dosages 60 min after KE administration (β = 4.9 µmol/L per 1 mmol/L D-BHB; 95% CI: 2.4–7.4; unadjusted *p* < 0.001; Fig. 5D). Unadjusted correlations suggested a positive correlation after 20 g (*p* = 0.04) and 40 g (*p* = 0.06) of KE; however, neither remained statistically significant after Holm-Bonferroni correction. In Cohort IV, D-BHB and D-BHB-Phe were moderately correlated after oral KS administration (Pearson’s *r* = 0.72, unadjusted *p* = 0.05); however, this correlation did not remain statistically significant after Holm-Bonferroni correction for multiple comparisons. No correlation was observed during intravenous KS infusion (Fig. 5D).

**Fig. 6:**
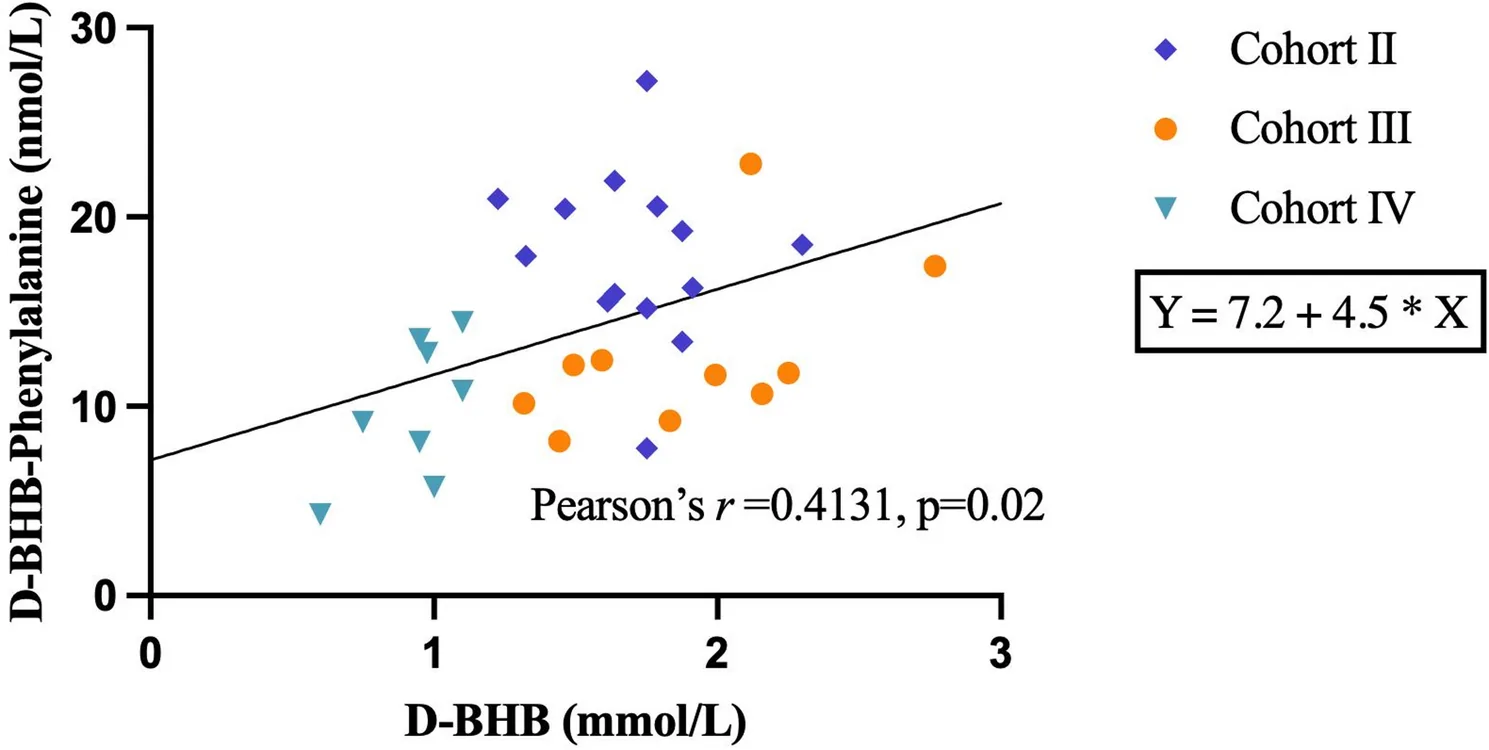
Relationship between mean D-BHB and D-BHB-phenylalanine concentrations aggregated across interventions involving orally induced ketosis (Cohorts II-IV). Each point represents one participant’s mean values across all timepoints (0–120 min) and, for Cohorts II and III, across all interventions with ketone supplements. A simple linear regression indicated a positive association between D-BHB and D-BHB-phenylalanine (regression equation shown in the figure: *Y = 7.2 + 4.5 × X*). D-BHB: D-β**-**hydroxybutyrate

Lastly, in Cohorts II-IV simple linear regression showed a positive association between mean D-BHB and D-BHB-phenylalanine concentrations (β = 4.52, 95% CI: 0.80–8.24, *p* = 0.02; Fig. 6). The model indicated that the D-BHB concentration explained 17.1% of the variation in the D-BHB-Phe concentration (R²=0.171, F (1, 30) = 6.17, *p* = 0.02). Pearson’s correlation showed a moderate positive correlation (*r* = 0.41, *p* = 0.02). However, after adjusting for cohort, the relationship was attenuated and no longer statistically significant (β = 3.32, 95%CI: -1.74–8.38, *p* = 0.19). Additionally, leave-one-cohort-out sensitivity analyses indicated that the overall association was strongly influences by cohort IV, as exclusion of this cohort abolished the relationship. In contrast, exclusion of cohorts II and III strengthened the association (Supplementary Table 2).

## Discussion

In this study, we investigated circulating concentrations of D-BHB-Phe in humans under various forms and degrees of ketosis. Across all cohorts, we consistently found negligibly low D-BHB-Phe concentrations at baseline and under placebo/control conditions. Plasma concentrations of D-BHB-Phe increased following a ketogenic diet and after oral administration of supplements containing both KE and KS. Also, D-BHB concentrations increased dose-dependently following KE administration at three different doses, and this pattern was mirrored by corresponding elevations in D-BHB-Phe. Lastly, oral KS administration induced substantially higher D-BHB-Phe concentrations compared with isoketotic intravenous KS delivery.

We confirmed our first hypothesis by showing that 21 days on a ketogenic diet elevated the D-BHB-Phe concentration in humans. Notably, this finding contrasts with a previous study reporting no effect of endogenous ketosis induced by a 48-h fast on the D-BHB-Phe concentration in both normal-weight or obese individuals [7], despite achieving D-BHB concentrations similar or higher than those observed in cohort I. However, this study reported substantially higher and more variable baseline BHB-Phe concentrations, particularly in the obese cohort, than observed in the current study, which could have hindered showing an effect. Additionally, although both the ketogenic diet and prolonged fasting induce ketosis, they differ metabolically; for example, during a ketogenic diet the splanchnic bed is continuously exposure to macronutrients, whereas such signals are absent during a prolonged fast. As such, metabolic context may be important in understanding D-BHB-Phe responses. The ketogenic diet has been found to reduce appetite in humans [12], yet the underlying mechanisms remain poorly defined and could involve direct effects of ketone bodies as well as downstream metabolites, such as D-BHB-Phe. Exogenous ketone supplementation has been shown to decrease the concentration of the orexigenic hormone ghrelin [13, 14], reduce gastric emptying [11], and dampen subjective appetite sensations [13, 14], but whether these effects are mediated by D-BHB or related metabolites such as D-BHB-Phe is unclear. The anorexigenic properties of D-BHB-Phe observed in mice [3] suggest that this metabolite could contribute to these appetite-suppressing effects. Upon peritoneal injections with D-BHB-Phe or Lac-Phe in mice, resulting in pharmacologically elevated plasma concentrations, activation of several hypothalamic and brainstem regions implicated in the regulation of energy balance, as well as circadian rhythm and cardiovascular control was observed [3]. Given this apparent central mechanism of action, D-BHB-Phe might also influence other metabolic outcomes linked to elevated D-BHB concentrations, such as improved cardiovascular function [15] and cognitive performance [16, 17]. Furthermore, D-BHB-Phe could contribute to the glucose-lowering effects of exogenous ketones [18], analogous to the improved glycemic regulation observed with Lac-Phe treatment in mice [5]. The molecular targets through which D-BHB-Phe exerts its physiological effects have, however, not yet been identified. As such, it remains unknow whether D-BHB-Phe and related BHB-amino acid conjugates engage established metabolite-sensing receptors or act through distinct receptor-mediated mechanisms. Clarifying the extent of these potential actions awaits future clinical investigations.

We also confirmed our second hypothesis by demonstrating that isocaloric doses of KS and KE increased D-BHB-Phe concentrations compared with placebo (water) in cohort II.

Cohort III was included to assess whether increases in the D-BHB concentration were associated with corresponding changes in D-BHB-Phe. Within this cohort, escalating KE doses produced progressive increases in D-BHB-Phe concentrations and an overall positive association across all KE dosages, reflecting a dose-dependent response to ketone exposure. Consistent with this, in Cohort IV, circulating D-BHB concentrations following oral KS administration were positively correlated with D-BHB-Phe levels; however, this correlation did not remain statistically significant after Holm–Bonferroni correction. In contrast, no correlation between systemic D-BHB and D-BHB-Phe was observed in Cohort II. Notably, across all cohorts, D-BHB-Phe concentrations only increased during states of ketosis, further supporting an association between D-BHB and D-BHB-Phe formation. Taken together, these findings suggest a generally positive, albeit variable, relationship between circulating D-BHB and D-BHB-Phe concentrations. A recently published study reported a clear and significant increase in D-BHB-Phe after KE ingestion in healthy, lean, young adults [6]. In this study, KE was administered on a body-weight basis, resulting in an average dose of approx. 55 g, substantially higher than the 10 to 40 g of KE administered in cohorts II and III, which may have enhanced the detectability of a D-BHB to D-BHB-Phe correlation. However, differences in metabolic context may also be important. Participants in that study were metabolically healthy, whereas participants in cohorts II and III were all diagnosed with type-2 diabetes, a condition associated with impaired metabolic flexibility. Accordingly, responses in cohort II and III may more closely resemble those observed in individuals with obesity in whom an uncoupled relationship between D-BHB and D-BHB-Phe has been reported [7]. Consistent with this interpretation, although systemic D-BHB was positively associated with D-BHB-Phe in the regression analysis of interventions inducing oral ketosis, systemic D-BHB explained only 17% of the variation in D-BHB-Phe, and the two metabolites correlated only moderately. However, the association was dependent on cohort and became non-significant upon excluding lower D-BHB concentrations (cohort IV). Collectively, these findings suggest that while D-BHB availability may contribute to D-BHB-Phe formation, circulating D-BHB alone is not a sufficient determinant of D-BHB-Phe. Instead, additional factors, such as metabolic state, splanchnic exposure to D-BHB, and hepatic clearance, are likely to play an important role. This may partly explain why the two exogenous ketone formulations (KE vs. KS) in cohort II produced comparable D-BHB-Phe concentrations despite three-fold higher circulating D-BHB levels following KE compared with KS. Notably, KS contains racemic sodium (DL)-3-hydroxybutyrate and is therefore directly exposed to the gastrointestinal tract and splanchnic circulation, whereas the KE, following hydrolysis, mainly in the gastrointestinal tract, liberates one equivalent of D-BHB and one equivalent of the ketone precursor D-1,3-butanediol, which, after absorption, is converted to D-BHB in the liver [19]. Accordingly, differences in gastrointestinal absorption, stereochemistry, and metabolic processing between KS and KE likely contribute to the divergent relationships observed between D-BHB and D-BHB-Phe. Overall, these findings indicate that the relationship between D-BHB and D-BHB-Phe is more complex than can be explained by systemic D-BHB concentrations alone.

Lastly, we confirmed our fourth hypothesis by showing that oral KS administration resulted in significantly higher D-BHB-Phe concentrations compared with isoketotic intravenous KS infusion, indicating that D-BHB availability in the splanchnic bed is critical for D-BHB-Phe formation. This interpretation aligns with high carnosine dipeptidase 2 (CNDP2) expression in gut and kidney tissue in mice [3] and RNA expression in the human gastrointestinal tract [20]. Compared with oral ketone interventions, D-BHB-Phe concentrations were only modestly increased after three weeks on a ketogenic diet and during intravenous KS administration, both of which bypass the gut lumen. In contrast, oral ketone supplements, presumably generating higher local D-BHB concentrations, raised D-BHB-Phe to ~ 40 nmol/L, whereas interventions with low gut exposure (placebo, intravenous KS, ketogenic diet; blue box in Fig. 5E) reached only ~ 5 nmol/L. These findings underscore the potential role of gastrointestinal CNDP2 and local D-BHB availability in BHB-Phe synthesis. Nonetheless, the physiological relevance of circulating D-BHB-Phe concentrations in humans remains unclear, as it is not yet known whether the nanomolar levels observed in vivo fall within a biologically active range.

The splanchnic-dependent pattern extends to other CNDP2-related metabolites. Elevated L-Lac-Phe concentrations after oral versus intravenous lactate administration [8] suggest that intestinal metabolism plays a key role in L-Lac-Phe formation. Consistent with this, Metformin-induced Lac-Phe likely reflects increased intestinal lactate production [21, 22]. Metformin treatment is associated with a modest but significant weight loss through multiple central and peripheral mechanisms [23]. The increase in Lac-Phe may represent another potential pathway for Metformin-induced weight loss. Whether medication with ketogenic effects, such as SGLT2 inhibitors, can induce a similar increase in D-BHB-Phe is currently unknown and warrants further investigations. If so, D-BHB-Phe could contribute as an additional mechanism to the weight loss induced by SGLT2 inhibitors beyond glucosuria [24]. However, as noted previously, evidence for appetite regulation and weight reduction by D-BHB-Phe and Lac-Phe is currently limited to studies in mice [3, 5]. While clinical trials on Lac-Phe are underway (ClinicalTrials.gov ID: NCT06743009), future studies should clarify whether D-BHB-Phe exerts similar effects in humans.

The findings of this study provide new insights into D-BHB-Phe metabolism in humans; however, several limitations should be acknowledged. This was an exploratory study utilizing plasma samples from four previous studies, none of which were originally powered for the outcomes assessed here. Although all studies were controlled, sample sizes were relatively small, and - except for Cohort I (ketogenic diet) - they examined the acute effects of BHB administration. Consequently, the impact of chronic administration on D-BHB-Phe remains unknown, as does the potential difference between metabolic responses to acute endogenous ketosis and the more prolonged endogenous ketosis induced in Cohort I. The cohorts also represented heterogeneous populations: Cohort I only included middle-aged individuals due to radiation exposure during positron emission tomography/computed tomography scans. Cohorts II and III enrolled people with type-2 diabetes. Cohort IV comprised healthy, young males. While the limited transcriptomic data available suggest broadly comparable CNDP2 expression across the gastrointestinal tract in individuals with type-2 diabetes and healthy controls, except for a significant down regulation in the ileocecal region [20], a recent study suggests an uncoupled relationship between D-BHB and D-BHB-Phe in individuals with obesity [7]. Whether the same applies to type-2 diabetes remains unknown. Study conditions differed as well, with MMT/OGTT performed in Cohorts II and III, whereas Cohorts I and IV were investigated during fasted conditions. It is unknown how these differences in study conditions influences D-BHB-Phe. Thus, cross-cohort comparisons should be interpreted cautiously. This is especially important to keep in mind when interpretating the linear regression on the aggregated data (Fig. 6), as population heterogeneity and differences in study conditions may contribute to residual variance. Our inference of splanchnic involvement in D-BHB-Phe synthesis is based on indirect evidence, as CNDP2 expression or activity was not directly measured. Although D-BHB-Phe suppresses appetite in mice, we did not assess subjective appetite sensations, and its physiological relevance for human appetite regulation remains unclear. Given the acute nature of the interventions, the small sample sizes, and baseline concentrations at or near the detection limit prior to each intervention exposure, no formal statistical testing for carryover was conducted. Lastly, methodological limitation is the use of a structurally related internal standard (d₅-Lac-Phe), which may not fully account for matrix effects specific to D-BHB-Phe. Despite this, we have no reason to believe that this limitation impacts the interpretation of our results.

## Conclusion

In conclusion, our findings demonstrate that D-BHB-Phe can be induced through both endogenous ketosis and ingestion of exogenous ketones and suggest that the splanchnic bed may be a key site of its synthesis. While the magnitude of this response varied across metabolically distinct cohorts, these results provide a foundation for future studies exploring the role of D-BHB-Phe in human appetite regulation and metabolic health.

## Supplementary Information

Below is the link to the electronic supplementary material.

Supplementary Material 1

## Data Availability

Data are available upon responsible request to the corresponding author.

## Abbreviations

BHB: Beta-hydroxybutyrate
KE: Ketone ester
KS: Ketone salt
OGTT: Oral glucose tolerance test
MMT: Mixed meal test
