# Supplementary material for: Ketosis elevates plasma concentrations of D-β-hydroxybutyryl-phenylalanine (BHB-Phe) in humans
Source: Nutr Metab (Lond). 2026 Jul 15;23:120. doi: 10.1186/s12986-026-01172-7 (PMC13651392; doi:10.1186/s12986-026-01172-7)
Supplement: Supplementary file 1 [file 12986_2026_1172_MOESM1_ESM.docx]

**Supplementary Methods.**

Liquid Chromatography Tandem Mass Spectroscopy

*Reagents*

Acetonitrile, methanol (MeOH), and formic acid (FA) were purchased from VWR International, LLC (Radnor, USA). Ultrapure water was produced in-house using a Milli-Q water system, (Millipore, Billerica, USA). D-BHB-Phe authentic standard was purchased from purchased from Enamine Ltd. (Kyiv, Ukraine) and d5-Lac-Phe (N-lactoyl-phenylalanine-d5) internal standard (IS) was purchased from Hello Bio Limited (Dunshaughlin, Ireland). Standards were dissolved in ultrapure water to 5 mM stock solutions and diluted and combined down to calibrator solutions at 0.2, 0.5, 1, 5, 10, 25 and 50 nM and quality control spike solutions at 0.5, 10 and 50 nM.

*Sample preparation for targeted analysis*

100 μL of plasma was aliquoted to which 100 μL of IS solution, and 200 μL of 100% MeOH was added for protein precipitation. Calibrator samples and quality control samples were prepared in parallel; 100 μL of ultra-pure water was aliquoted to which 100 μL of IS solution, 100 μL of calibrator solution/quality control solution, and 100 μL of 100% MeOH was added. All sample preparation was carried out on ice.

The extraction suspension was shaken at room temperature for 5 min at 1000 RPM, chilled at 4 °C for 10 min, and then centrifuged (15 x g, 10 min, room temperature). Supernatants were transferred to a 96-well plate and dried at 40°C using a Nitrogen evaporator. Dried samples were reconstituted in 300 μL of 0.1% FA and transferred to MS vials for LC-MS/MS analysis.

*Targeted analysis of plasma samples (LC-MS/MS analysis)*

Targeted analysis was conducted using a SCIEX Exion LC™ system (SCIEX, Framingham, MA, USA) coupled to a QTRAP® 6500+ mass spectrometer (SCIEX, Framingham, MA, USA). Chromatographic separation was achieved on an ACQUITY UPLC HSS T3 C18 column (2.1 × 100 mm, 1.8 μm; Waters, Dublin, Ireland). The autosampler was maintained at 15 °C, and 10 μL of each sample was injected. The column temperature was set to 45 °C, with a constant flow rate of 0.35 mL/min.

The mobile phases consisted of water containing 0.1% (v/v) formic acid (A) and a 1:1 (v/v) mixture of methanol and acetonitrile supplemented with 0.1% (v/v) formic acid (B). Analytes were eluted using a 15 min gradient as follows: 0% B from 0–2 min; 0–40% B from 2–6 min; 40–60% B from 6–9 min; 60–88% B from 9–10 min; 88–100% B from 10–11 min; held at 100% B from 11–12 min; returned to 0% B from 12–13 min; and re-equilibrated at 0% B from 13–15 min. Detection was performed using multiple reaction monitoring (MRM) with electrospray ionization in negative mode (ESI−). The IonSpray voltage was set to −4.0 kV, collision gas was set to medium, and the dwell time was 25 ms per transition. MRM transitions for D-BHB-Phe and the internal standard, d₅-Lac-Phe, were selected through independent optimization of precursor and product ions to ensure sensitivity and selectivity under the applied LC–MS/MS conditions. Product ions were selected from fragmentation spectra based on signal intensity and specificity. Due to differences in the acyl moieties, D-BHB-Phe and d₅-Lac-Phe exhibit distinct fragmentation pathways; therefore, MRM transitions were not selected to reflect identical product ions but rather to ensure optimal detection of each compound. The internal standard was used to correct for variability in sample preparation and instrument response. The precursor–product ion transitions and corresponding optimized MRM settings (collision energy, declustering potential, and entrance potential) are listed in Supplementary table 1.

**Supplementary table 1: The precursor–product ion transitions and corresponding optimized MRM settings.**

|  | **MRM transitions (m/z)** | **Collision energy (eV)** | **Declustering potential (V)** | **Entrance potential (V)** |
| --- | --- | --- | --- | --- |
| D-BHB-Phe | 250.11 → 57.9 | -38 | -22 | -6 |
| D-BHB-Phe | 250.11 → 147.1 | -33 | -22 | -6 |
| D-BHB-Phe | 250.11 → 164.0 | -20 | -22 | -6 |
| d_5_-Lac-Phe | 241.1 → 88.1 | -21 | -75 | -6 |
| d_5_-Lac-Phe | 241.1 → 96.1 | -29 | -75 | -6 |
| d_5_-Lac-Phe | 241.1 → 151.8 | -21 | -75 | -6 |

Data acquisition was performed using Analyst 1.5 software (AB Sciex, Concord, Ontario, Canada), and quantitative analysis was conducted using MultiQuant™ 3.0 software (AB Sciex, Concord, Ontario, Canada). Quantification of D-BHB-Phe was based on integrated peak areas normalized to d_5_-Lac-Phe area. A seven-point calibration curve was generated by linear regression of the analyte-to-IS peak area ratio against actual analyte concentrations.

Supplementary Figure 5 provides representative LC–MS/MS chromatograms illustrating the detection of D‑BHB‑Phe in a 100 nM standard and in unspiked human plasma.

**Supplementary Results**

**Supplementary Table 2: Regression analyses assessing the association between D‑BHB and D‑BHB‑phenylalanine.**

|  | **β** | **p** | **R²** |
| --- | --- | --- | --- |
| Raw regression | 4.5 | 0.02 | 0.17 |
| Adjusted for cohort | 3.3 | 0.19 | 0.44 |
| Excluding cohort 2 | 3.9 | 0.02 | 0.32 |
| Excluding cohort 3 | 7.7 | 0.003 | 0.36 |
| Excluding cohort 4 | 0.4 | 0.9 | 0.001 |

Regression coefficients (β), p-values, and coefficients of determination (R²) are shown for the unadjusted model, model adjusted for cohort, and leave-one-cohort-out sensitivity analyses.

**Supplementary Figures**

## Enrollment

Assessed for eligibility (n=12)

Included (n=12)

## Allocation

Ketogenic diet (n=6)

Standard diet (n=6)

Drop-out (n=1)

Standard diet

Ketogenic diet

## Analysis

Analyzed in current study (n=10)

Analyzed in original study (n=11)

No available samples (n=1)

**Supplementary figure 1: Cohort I Consort Flow Diagram**

Assessed for eligibility (n = 66)

**Enrollment**

Excluded (n = 52)

- Not meeting inclusion criteria (n = 36)
- Declined to participate (n = 16)

Randomized (n = 14)

**Allocation**

**Day 1**

KS (n = 5)

KE (n = 5)

PLC (n = 4)

KS

**Day 2**

KE

PLC

PLC

**Day 3**

KS

KE

**Analysis**

Analyzed (n = 14)

# **Supplementary Figure 2: Cohort II Consort Flow Diagram**

KS: ketone salt, KE: ketone ester, PLC: placebo.

Assessed for eligibility (n = 20)

**Enrollment**

Excluded (n = 10)

- Not meeting inclusion criteria (n = 1)
- Declined to participate (n = 9)

Randomized (n = 10)

**Allocation**

Interventions received in random order (n = 10):

i) 0 g KE 30 minutes before an OGTT (PLC)
ii) 10 g KE 30 minutes before an OGTT
iii) 20 g KE 30 minutes before an OGTT
iv) 40 g KE 30 minutes before an OGTT
v) 20 g KE 0 minutes before an OGTT
vi) 20 g KE 60 minutes before an OGTT

Not completed:

- Intervention v (n = 2)

**6 study days**

**Analysis**

Interventions analysed in the current study (n=10)

i) 0g KE 30 min before an OGTT (Plc)
ii) 10g KE 30 min before an OGTT
iii) 20 g KE 30 min before an OGTT
iv) 40 g KE 30 min before an OGTT

# **Supplementary Figure 3: Cohort III Consort Flow Diagram**

KE: ketone ester, PLC: placebo, OGTT: oral glucose tolerance test.

## Enrollment

Assessed for eligibility (n=9)

Not included

- Declined to participate (n=1)

Included (n= 8)

Analysed (n=8)

## Analysis

Supplementary Figure 4: Cohort IV Consort Flow Diagram

**Supplementary Figure 5:** Representative LC–MS/MS chromatograms of D-BHB-Phe. Chromatogram obtained from a 100 nM standard (left). Chromatogram from unspiked human plasma (right).
